# Electric field–induced inflammatory and angiogenic responses in rat female reproductive tissues: evidence for early extracellular matrix remodeling

**DOI:** 10.1007/s00418-026-02470-0

**Published:** 2026-03-27

**Authors:** Ozlem Ozmen, Halil Asci, Oznur Kolay, Elif Nur Selcuk, Duygu Yuksel, Selcuk Comlekci, Pinar Karabacak

**Affiliations:** 1 Department of Pathology, Faculty of Veterinary Medicine, Burdur Mehmet Akif Ersoy University, Burdur, Türkiye; 2 Department of Pharmacology, Faculty of Medicine, Suleyman Demirel University, Isparta, Türkiye; 3 Department of Medical Pharmacology, Institute of Health Sciences, Suleyman Demirel University, Isparta, Türkiye; 4 Department of Mechanical Engineering, Institute of Science and Technology, Suleyman Demirel University, Isparta, Türkiye; 5 Medical Services and Techniques Department, Gumuşhane Vocational School of Health Services, Gumushane University, Gumushane, Türkiye; 6 Department of Electrical and Electronics Engineering, Faculty of Engineering, Suleyman Demirel University, Isparta, Türkiye; 7 Department of Anesthesiology and Critical Care, Faculty of Medicine, Suleyman Demirel University, Isparta, Türkiye

**Keywords:** Electric field, Ovary, Tumor necrosis factor alpha, Vascular endothelial growth factor, Tissue remodeling

## Abstract

High-intensity electric fields (EFs) are increasingly encountered in occupational and environmental settings, raising concerns regarding their potential biological effects on hormonally responsive organs. However, the cellular and molecular responses of female reproductive tissues to short-term exposure to high-voltage EFs remain insufficiently characterized. This study aims to investigate EF-associated histopathological and immunohistochemical alterations in major female reproductive organs. Forty adult female Wistar albino rats were randomly assigned to five experimental groups (*n* = 8 per group): a control group (0 min) and four EF-exposed groups subjected to a nominal electric field intensity of 10 kV/m EF for 1, 5, 15, or 30 min using a custom-designed parallel-plate exposure system. The field intensity was verified at predefined measurement points within the exposure setup. Ovaries, uterus, and uterine tubes were examined using hematoxylin–eosin staining and semi-quantitative histopathological scoring. Immunohistochemical analyses were performed to evaluate the expression of tumor necrosis factor-α (TNF-α), vascular endothelial growth factor (VEGF), and osteonectin as markers of inflammation, angiogenesis, and extracellular matrix remodeling. Exposure to the electric field was associated with time-dependent histopathological alterations, including tissue edema, hemorrhage, epithelial degeneration, and leukocyte infiltration. The most pronounced ovarian and uterine changes were observed in the 30-min exposure group, whereas the uterine tubes exhibited comparatively milder structural alterations. Immunohistochemical analysis demonstrated increased TNF-α and VEGF expression in higher-exposure groups, suggesting activation of inflammatory and angiogenic pathways. Osteonectin expression was elevated in all examined reproductive tissues and was also detected in regions without overt morphological damage, indicating its potential sensitivity to early tissue stress responses associated with EF exposure. Significant correlations were observed between histopathological injury scores and immunohistochemical marker expression levels. Overall, short-term exposure to high-intensity electric field was associated with inflammatory signaling, angiogenic responses, and extracellular matrix remodeling in female reproductive tissues in this experimental model. These findings provide histological and immunohistochemical evidence of tissue responses to EF exposure and underscore the need for further studies incorporating comprehensive exposure characterization and additional molecular endpoints to better define potential reproductive health implications to high-voltage electric fields.

## Introduction

The female reproductive system is highly sensitive to both biochemical and physical stimuli, including electric and electromagnetic fields (Athar et al. 2024; Topsakal et al. 2024). Depending on field intensity, exposure duration, and tissue characteristics, external electric fields (EFs) may influence cellular and tissue responses such as membrane polarization, ion channel modulation, oxidative stress, inflammatory signaling, and apoptosis (Kadan-Jamal et al. 2025). While low- to moderate-intensity fields have been reported to support certain regenerative or bioelectrical regulatory processes, higher field intensities in experimental systems have been associated with cellular stress responses and functional alterations (Zhao et al. 2006; Li et al. 2023; Ozcan et al. 2025).

Experimental studies using rodent models have demonstrated that electromagnetic field (EMF) exposure can induce structural and biochemical changes in female reproductive tissues. For example, exposure to 900 MHz EMFs increased endometrial apoptosis and oxidative stress markers in rats (Oral et al. 2006; Alchalabi et al. 2016), whereas low-frequency EMFs have been associated with ultrastructural follicular damage and apoptotic alterations suggestive of disrupted folliculogenesis (Roushangar and Rad 2007; Roshangar et al. 2014). Additional studies reported alterations in ovulation dynamics, reduced corpus luteum formation, prolonged estrous cycles, and oocyte degeneration following EMF exposure (Jung et al. 2007; Gye and Park 2007; Khaki et al. 2011). Despite these findings, comparatively fewer studies have examined the biological effects of high-intensity electric fields, particularly at the histopathological and immunohistochemical levels.

Several mechanisms have been proposed to explain, EF-related cellular responses. High-intensity electric fields may influence membrane potential stability, calcium influx, and the generation of reactive oxygen species (ROS), thereby contributing activation of inflammatory and apoptotic signaling pathways (Nuccitelli et al. 2013; Duncan et al. 2016). Hormonally regulated and highly vascularized tissues—such as the ovaries, uterus, and uterine tubes—may be particularly responsive to such stressors due to their dynamic cellular turnover and vascular activity (Agarwal et al. 2012; Okatan et al. 2018). Among the mediators potentially involved in these processes are tumor necrosis factor-α (TNF-α) and vascular endothelial growth factor (VEGF), which regulate inflammation, angiogenesis, and extracellular matrix remodeling (Ruan et al. 2015). Another relevant marker, osteonectin (SPARC), a matricellular protein associated with tissue remodeling and angiogenesis that is often upregulated in tissues exposed to mechanical or biochemical stress (Bradshaw 2012). Although osteonectin expression has been described in reproductive tissues and ovarian pathology (Brown et al. 1999; Cain et al. 2025), its potential modulation following exposure to high-voltage electric fields has not been systematically evaluated.

From a physical standpoint, electric fields can exist independently of magnetic fields and may arise from static charge accumulation or voltage gradients (Bering et al. 1998). Static and high-voltage EFs are generated by both natural and anthropogenic sources, including atmospheric phenomena, high-voltage transmission systems, and certain industrial installations (Maruvada 2012; Petri et al. 2017). In some occupational environments, EF intensities approaching several kilovolts per meter have been reported in proximity to high-voltage infrastructure, corresponding to the upper range of public or occupational exposure limits discussed in international safety guidelines (ICNIRP 2010).

Despite increasing interest in the potential biological implications of EF exposure, detailed histopathological and molecular investigations focusing specifically on high-intensity electric fields remain limited. Therefore, the present study aims to investigate the time-dependent effects of exposure to a nominal 10 kV/m EF on female rat ovaries, uterus, and uterine tubes using a controlled experimental exposure system. By evaluating TNF-α, VEGF, and osteonectin expression together with histopathological alterations, the study seeks to provide insight into inflammatory, angiogenic, and extracellular matrix–related tissue responses associated with short-term EF exposure.

## Material and methods

### EF exposure design

A 10 kV/m EF intensity was selected based on previous studies demonstrating that fields of this magnitude can induce biologically relevant cellular and tissue responses without causing detectable thermal injury or necrosis (Ozcan et al. 2025; Akın et al. 2025). This exposure level approaches the range in which electromechanical perturbations of cellular structures may occur, enabling investigation of inflammatory signaling pathways, vascular reactivity, and extracellular matrix remodeling while minimizing the risk of irreversible electrothermal damage (Zhao et al. 2006; Okatan et al. 2018; Li et al. 2023). Despite accumulating evidence indicating that electric fields can influence cellular processes, comprehensive histopathological and molecular investigations of EF exposure in reproductive tissues remain limited (Kirson et al. 2004, 2007).

The selected EF intensity also falls within the upper range of environmental and occupational electric field exposures reported in proximity to high-voltage power systems and certain biomedical applications, including electrotherapy and pulsed-field technologies, which may transiently generate comparable local field strengths (WHO 2007; Yarmush et al. 2014). Therefore, the applied field strength represents a biologically effective and translationally relevant exposure scenario for evaluating potential tissue responses, particularly in hormonally responsive and highly vascularized reproductive organs such as the ovary and uterus.

**Fig. 1:**
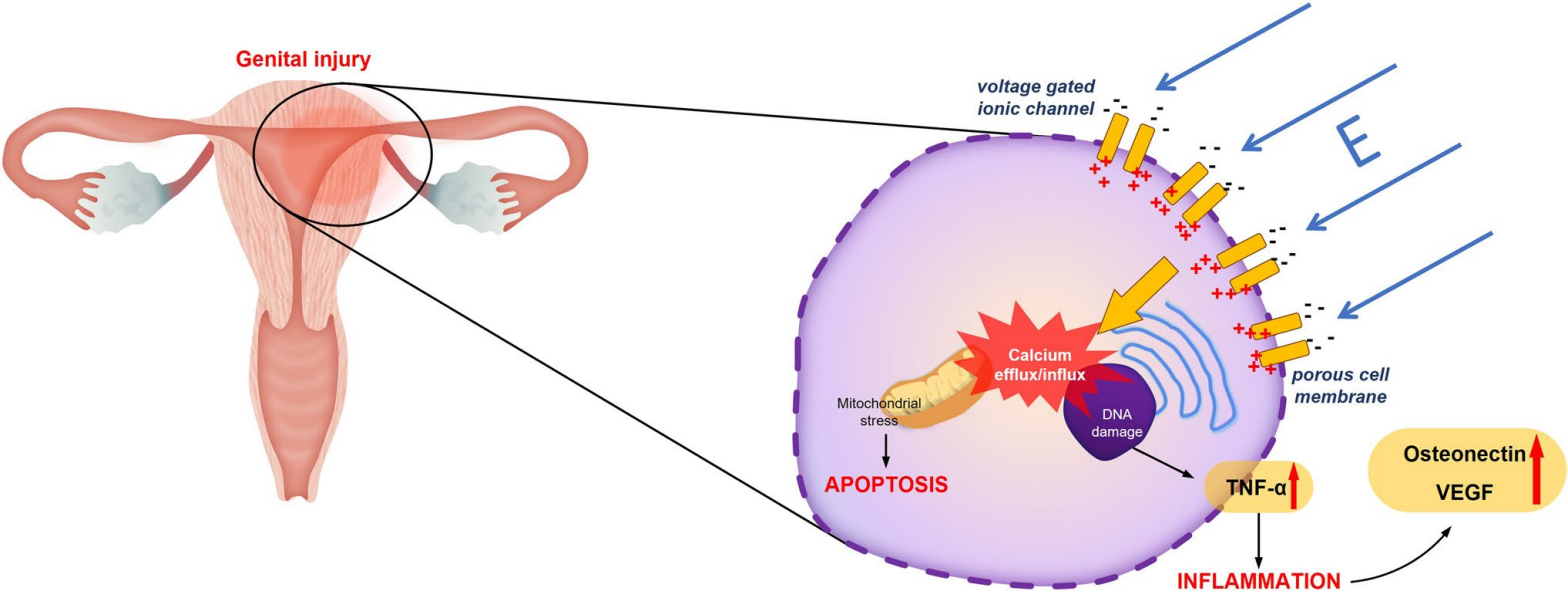
Schematic representation of potential cellular and tissue-level effects of high-intensity electric field exposure on the female reproductive tract. *EF* electric field, *TNF-α* tumor necrosis factor-alpha, *VEGF* vascular endothelial growth factor

EF exposure was performed using a custom-designed high-voltage parallel-plate system developed to produce a controlled and stable EF environment (Fig. 1). The apparatus consisted of two galvanized stainless-steel plates (50 cm × 100 cm, 1 mm thick, rounded corners) positioned horizontally at a fixed separation distance of 0.55 m and connected to a custom-built direct current (DC) high-voltage power supply (maximum 5.5 kVdc). Electrical connections were positioned centrally on each plate to minimize local distortion of the field distribution, and the plates were electrically isolated from the ground plane using on-conductive supports.

**Fig. 2:**
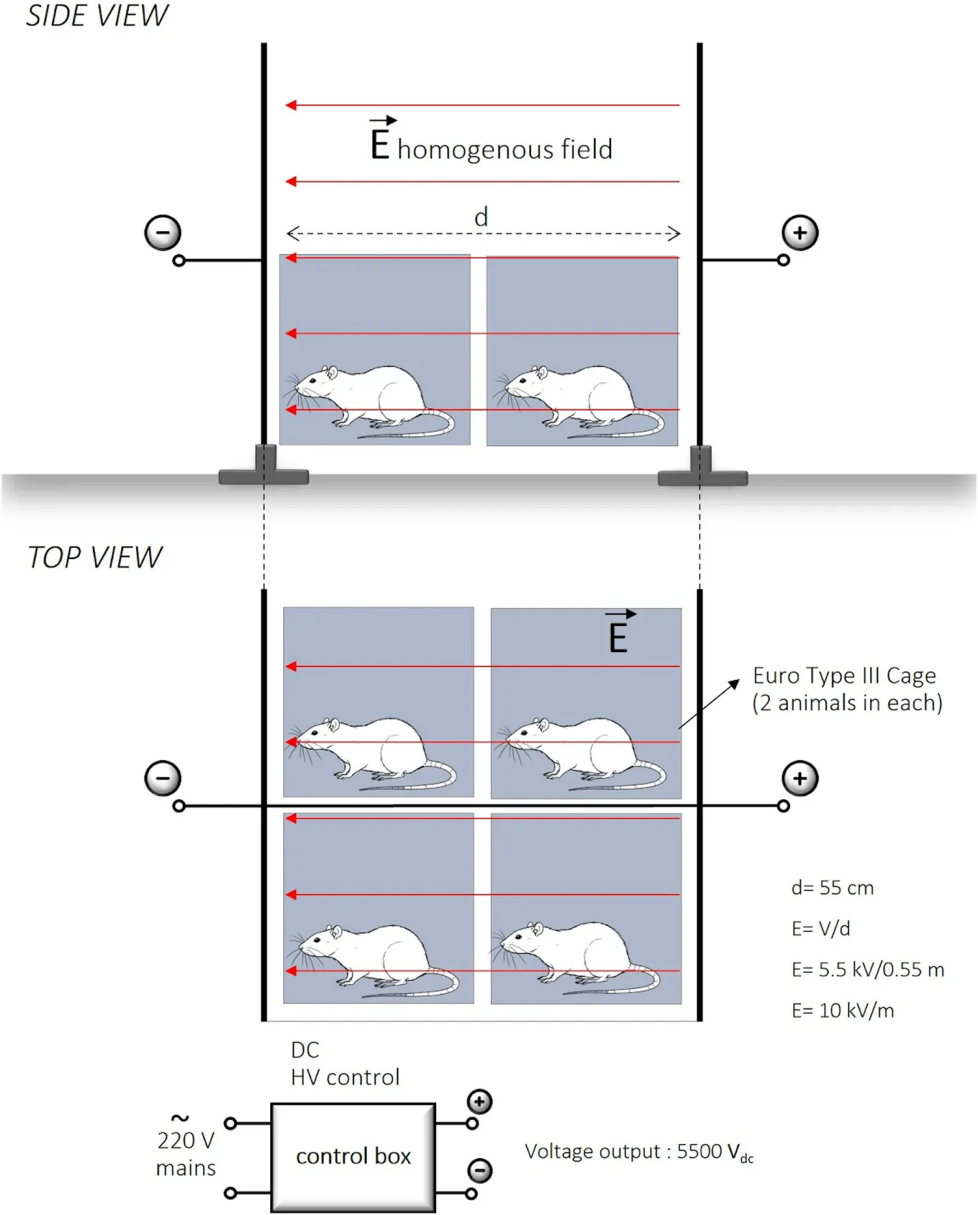
Schematic diagram of the experimental apparatus and electrical configuration used to generate a controlled high-intensity electric field for in vivo exposure of female rats. The setup ensures stable EF application across the central exposure region while allowing unrestrained animal movement

Animal cages (26 cm × 43 cm × 15 cm, nonconductive material) were placed centrally between the plates. Cages were spaced approximately 50 cm apart to standardize cage positioning within the exposure region and to reduce potential inter-cage perturbation of the electric field (Fig. 2). Animals were not physically restrained and were allowed to move freely within the cages during exposure to minimize stress-related neuroendocrine and inflammatory confounders. Animal behavior was continuously monitored to prevent excessive contact with conductive components of the experimental setup.

Comprehensive spatial mapping of the EF distribution across the entire cage volume was not performed. Instead, EF intensity was verified at representative positions within the central exposure region using a calibrated electric field meter under surrogate loaded conditions designed to approximate the dielectric presence of animals. Measurements were obtained at three predefined points approximately 10–15 cm above the cage floor, corresponding to the typical vertical position of the animals’ bodies during exposure.

The measured EF values ranged between 9.6 and 10.4 kV/m (mean ± standard deviation (SD): 10.0 ± 0.3 kV/m), confirming that the nominal exposure intensity was maintained within a narrow range around the target value in the central exposure region. These measurements were performed using the same geometric configuration as that employed in the experiments (parallel plates with centrally positioned cages and unrestrained animals). Because the animals were free to move within the cages during exposure, the reported measurements should be interpreted as nominal field values characterizing the central exposure zone rather than a fully resolved spatial field distribution.

In addition, the electrode dimensions were substantially larger than the cage footprint, a configuration commonly used in parallel-plate exposure systems to minimize edge-related field distortions and to stabilize the electric field in the central exposure region. Under such conditions, the central zone between the electrodes is expected to exhibit relatively stable field characteristics compared with peripheral regions. Consequently, animals positioned within the middle portion of the apparatus were exposed to comparable nominal EF conditions during the experimental sessions.

### Animals and experimental design

All experimental procedures were conducted in compliance with the Animal Research: Reporting of In Vivo Experiments (ARRIVE) 2.0 guidelines and internationally accepted ethical standards for animal research. The study protocol was approved by the Suleyman Demirel University Animal Experiments Local Ethics Committee (approval no. 18.09.2025–09/622). All procedures adhered to the principles of the 3Rs (replacement, reduction, and refinement) to minimize animal use and suffering. Animals were monitored daily for signs of distress, including reduced mobility, abnormal posture, respiratory difficulty, or body weight loss exceeding 20%. No adverse effects were observed throughout the study.

Sample size was determined using G*Power software (version 3.1.9.7) with the parameters *α* = 0.05, power (1 – *β*) = 0.90, and an effect size of 0.32, yielding a required sample size of eight animals per group. A total of forty adult female Wistar albino rats (12–14 weeks old; body weight 250–300 g) were obtained from the Experimental Animal Research Center of Suleyman Demirel University.

All female animals were age-matched and maintained under controlled environmental conditions to minimize physiological variability. Rats were housed in individually ventilated cages at a temperature of 22 °C ± 2 °C and relative humidity of 55–65%, under a 12-h light/12-h dark cycle, with ad libitum access to standard pellet chow and water. Before the initiation of the experimental procedures, all animals were acclimatized to the housing conditions for 1 week.

Animals were randomly allocated to the experimental groups to reduce potential allocation bias. Estrous cycle synchronization was not performed before EF exposure. However, all animals were selected within a similar age range and reproductive maturity stage to reduce potential variability. Because the primary aim of the study was to evaluate general in vivo tissue responses to short-term electric field exposure, random group allocation under standardized housing conditions was considered an appropriate approach to minimize potential cycle-related confounding effects.

Following acclimatization, animals were randomly assigned to five experimental groups (*n* = 8 per group) according to EF exposure duration: group I, control (0 min); group II, 1 min; group III, 5 min; group IV, 15 min; and group V, 30 min. Rats in the exposure groups were subjected to a single session of a nominal electric field intensity 10 kV/m generated by a custom-designed parallel-plate EF exposure system (Fig. 1). The field intensity was verified at predefined measurement points within the exposure setup. During exposure, animals were placed individually in a nonconductive cage positioned within the electrode system and were allowed to move freely without physical restraint. Control animals were handled under identical conditions but without activation of the electric field.

All animals remained within the experimental setup for a total duration of 30 min to ensure consistent environmental exposure across groups. In the control group, the device remained inactive for of the entire 30-min period. In the exposure groups, the electric field was activated for the designated exposure durations (1, 5, 15, or 30 min), after which the device was switched off while the animals remained in the setup for the remainder of the 30-min period.

After completion of the exposure protocol, all animals were monitored for vital signs, behavioral changes, and general well-being before euthanasia and tissue collection for histological and immunohistochemical analyses. Each animal underwent a single exposed session, after which the experimental protocol was completed.

### Histopathological examinations

Euthanasia was performed by decapitation under deep anesthesia induced by an intraperitoneal injection of xylazine (10 mg/kg; Xylazinbio 2%, Bioveta, Ivanovice na Hané, Czech Republic) and ketamine (90 mg/kg; Ketalar, Pfizer, İstanbul, Türkiye), in accordance with institutional and internationally accepted animal welfare guidelines. Adequate anesthetic depth was confirmed by the absence of pedal withdrawal and corneal reflexes before decapitation.

A comprehensive necropsy was subsequently performed, and tissues of the female reproductive system, including the ovaries, uterus, and uterine tubes, were carefully excised. Tissue samples were immediately fixed in 10% neutral buffered formalin and processed using a fully automated tissue processor (Leica ASP300S, Leica Microsystems, Wetzlar, Germany). Following routine dehydration and clearing procedures, the samples were embedded in paraffin wax and sectioned at a thickness of 5 μm using a rotary microtome (Leica RM2155, Leica Microsystems, Wetzlar, Germany).

All sections were stained with hematoxylin and eosin (H&E) (Sigma-Aldrich, cat. no. HT110132) and examined under a light microscope. Histological evaluations were independently performed by two experienced histopathologists affiliated with separate external institutions, both of whom were blinded to the experimental group allocations to minimize observational bias. Histopathological alterations in the ovary, uterus, and uterine tubes were assessed using a semiquantitative scoring system designed to provide standardized and reproducible evaluation of tissue injury. The evaluated parameters included vascular changes (hyperemia and hemorrhage), interstitial edema, inflammatory cell infiltration, and structural tissue damage (including cellular degeneration and epithelial loss). These features were defined according to established histopathological criteria. Each parameter was graded on a four-point ordinal scale based on lesion extent and severity and distribution (0 = absent, 1 = mild, 2 = moderate, 3 = severe), as summarized in Table 1.

**Table 1:** Parameters to scoring and characterize EF-induced tissue damage for each organ (ovary, uterus and uterine tube)

Histological criteria	Scores	Severity	Criterion
Vascular congestion (hyperemia)/edema/ hemorrhage/inflammatory cell infiltration/epithelial degeneration	0	Absent	Normal
	1	Mild	Lesions affecting less than 25% of the field
	2	Moderate	Lesions affecting approximately 26–50% of the field
	3	Severe	Lesions affecting more than 51% of the field

*EF* electric field

### Semi-quantitative scoring system

Histopathological alterations in the ovary, uterus, and uterine tube tissues were assessed using a semi-quantitative scoring system by two board-certified histopathologists who were independently blinded to the experimental group assignments. For each specimen, an overall tissue damage score was calculated by summing the scores of all evaluated histopathological parameters, ensuring standardized and reproducible assessment of tissue injury.

For each section, at least five randomly selected microscopic fields were examined at ×400 magnification using a light microscope (Olympus CX43, Tokyo, Japan). All scoring was performed manually, and the results were independently verified by a second blinded observer to confirm interobserver consistency. This systematic and blinded approach enabled robust comparison of tissue injury severity among experimental groups and facilitated correlation with immunohistochemical marker expression.

### Immunohistochemical examinations

Immunohistochemical staining was performed on selected sections of the female reproductive system to evaluate the expression of Osteonectin/SPARC (SPARC antibody, #DF6503; Affinity Bioscience, Canada), vascular endothelial growth factor (VEGF; VEGFA antibody, #AF5131; Affinity Bioscience, Canada), and tumor necrosis factor-alpha (TNF-α; anti-TNF-α recombinant antibody [RM1005], ab307164; Abcam, Cambridge, UK). After deparaffinization, rehydration, and heat-induced antigen retrieval, tissue sections were incubated with the respective primary antibodies at a dilution of 1:100 for 60 min at room temperature.

Immunodetection was carried out using a micro-polymer-based secondary antibody system (Mouse and Rabbit Specific HRP/DAB IHC Detection Kit; ab236466, Abcam, Cambridge, UK) according to the manufacturer’s instructions. This approach eliminates the need for a streptavidin–biotin complex and reduces background staining associated with endogenous biotin. Following incubation with the HRP-conjugated secondary reagent, immunoreactivity was visualized using diaminobenzidine (DAB) as the chromogen. Sections were then counterstained with Harris hematoxylin (Sigma-Aldrich, cat. no. HHS32), rinsed thoroughly under running tap water, and dehydrated through graded of ethanol series, cleared in xylene and coverslipped using Entellan mounting medium (catalogue no. 107961, Sigma-Aldrich, USA). Prepared slides were subsequently examined under a light microscope for semiquantitative analysis of marker expression.

### Image analysis and quantification

Appropriate positive and negative controls were included for each immunohistochemical marker. Osteosarcoma tissue served as a positive control for osteonectin, VEGF, and TNF-α immunoreactivity, whereas negative controls were prepared by omitting the primary antibody. All histological and immunohistochemical evaluations were performed in a blinded manner.

Sections were examined using a light microscope (Olympus CX43, Tokyo, Japan), and images were captured with a calibrated digital camera (Olympus DP27) using Plan-Apochromat objectives at ×10 and ×40 magnifications under standardized optical settings.

Immunohistochemically stained sections were evaluated through a combination of manual microscopic assessment and digital image analysis. For each marker (osteonectin, TNF-α, and VEGF), the percentage of positively stained cells was determined by counting 100 cells in five randomly selected high-power fields (×400 magnification) per tissue section. Quantitative analysis was performed using ImageJ software (version 1.53a; National Institutes of Health, Bethesda, MD, USA), applying color deconvolution and thresholding algorithms to isolate DAB-positive signals. Immunoreactivity was expressed as the ratio of positively stained area to total field area.

All images were analyzed under identical acquisition and processing parameters to ensure comparability. Measurements were independently performed by two investigators blinded to group allocation, and the mean values were used for statistical analysis. Interobserver reliability was assessed using intraclass correlation coefficients (ICCs), and discrepancies exceeding 10% were jointly re-evaluated. This methodology provided reproducible and objective quantification of immunohistochemical marker expression across experimental groups.

### Statistical analysis

Histopathological alterations were assessed using a semi-quantitative ordinal scoring system (0–3), were treated as nonparametric data. Intergroup comparisons were performed using the Kruskal–Wallis test, followed by Dunn’s multiple comparison post hoc test when a significant overall difference was detected.

Immunohistochemical (IHC) data, expressed as the percentage of positively stained cells, were treated as continuous variables. Normality of distribution was assessed using the Shapiro–Wilk test (p > 0.05), and group comparisons were subsequently conducted using one-way analysis of variance (ANOVA). When a significant main effect was detected, Tukey’s post hoc test was applied for multiple pairwise comparisons.

All statistical analyses were performed using GraphPad Prism software (GraphPad Software Inc., San Diego, CA, USA), and statistical significance was defined as *p* < 0.05.

## Results

### Histopathological findings

Histological evaluation of ovarian tissues revealed a clear time-dependent increase in structural damage following exposure to high-voltage (10 kV/m) EFs. Animals in different estrous phases were distributed across groups; therefore, comparisons were primarily conducted among animals in the same estrous stage. Lesions absent in control animals of the corresponding phase were attributed to EF exposure. Representative micrographs were obtained from animals in the diestrus phase.

The control group exhibited preserved ovarian architecture, with regularly distributed follicles, intact stromal organization, and minimal vascular congestion. The 1- and 5-min exposure groups showed no significant alterations in follicular morphology or stromal integrity. In contrast, the 15-min exposure group displayed focal vascular dilation and mild hyperemia. Structural damage was most pronounced in the 30-min group, which exhibited marked hemorrhage, stromal edema, increased leukocyte infiltration, and degeneration of follicular structures. Primordial, primary, and secondary follicles appeared shrunken, and the stromal matrix exhibited loosening and vacuolization.

**Fig. 3:**
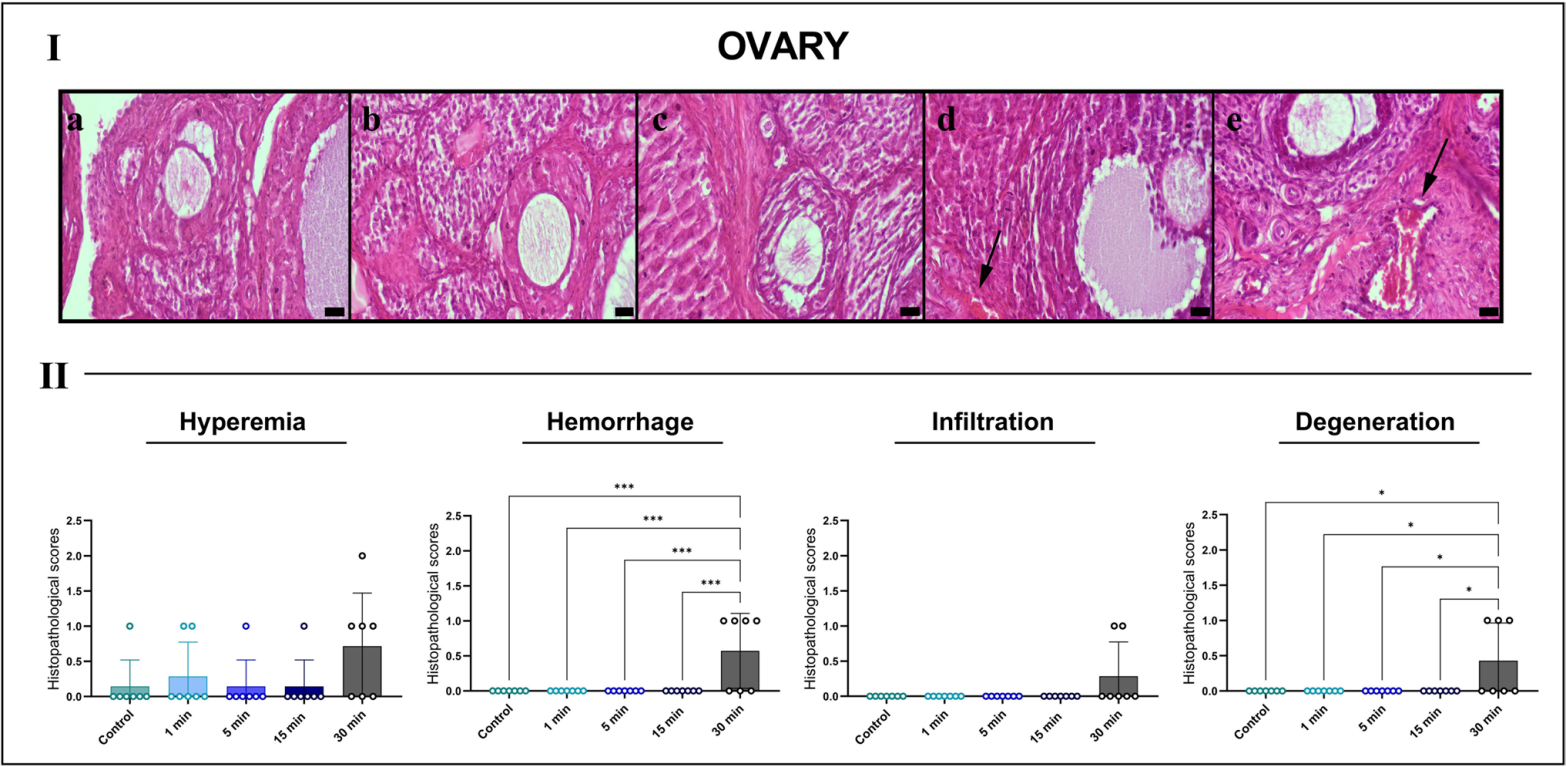
Representative HE-stained sections of rat ovarian tissues across experimental groups (I) and semi-quantitative histopathological scoring (II). **A** Control group displays normal follicular architecture, intact stroma and minimal vascular changes. **B** 1-min and **C** 5-min exposure groups show preserved ovarian morphology. **D** 15-min group exhibits early vascular hyperemia and mild interstitial edema (arrow). **E** 30-min EF exposure induces pronounced hyperemia (arrow) and stromal degeneration. Scale bars, 20 μm. *Quantitative scoring graphs indicate significant increases in hemorrhage (***p < 0.001) and stromal degeneration (*p < 0.05) in the 30-min group, analyzed using Kruskal–Wallis test with Dunn’s post hoc comparison

Semi-quantitative analysis confirmed significant increases in hemorrhage (***p < 0.001) and stromal degeneration (*p < 0.05) in the 30-min group compared with the control and shorter exposure groups, indicating substantial EF-induced ovarian injury likely mediated by inflammatory and microvascular mechanisms (Fig. 3).

**Fig. 4:**
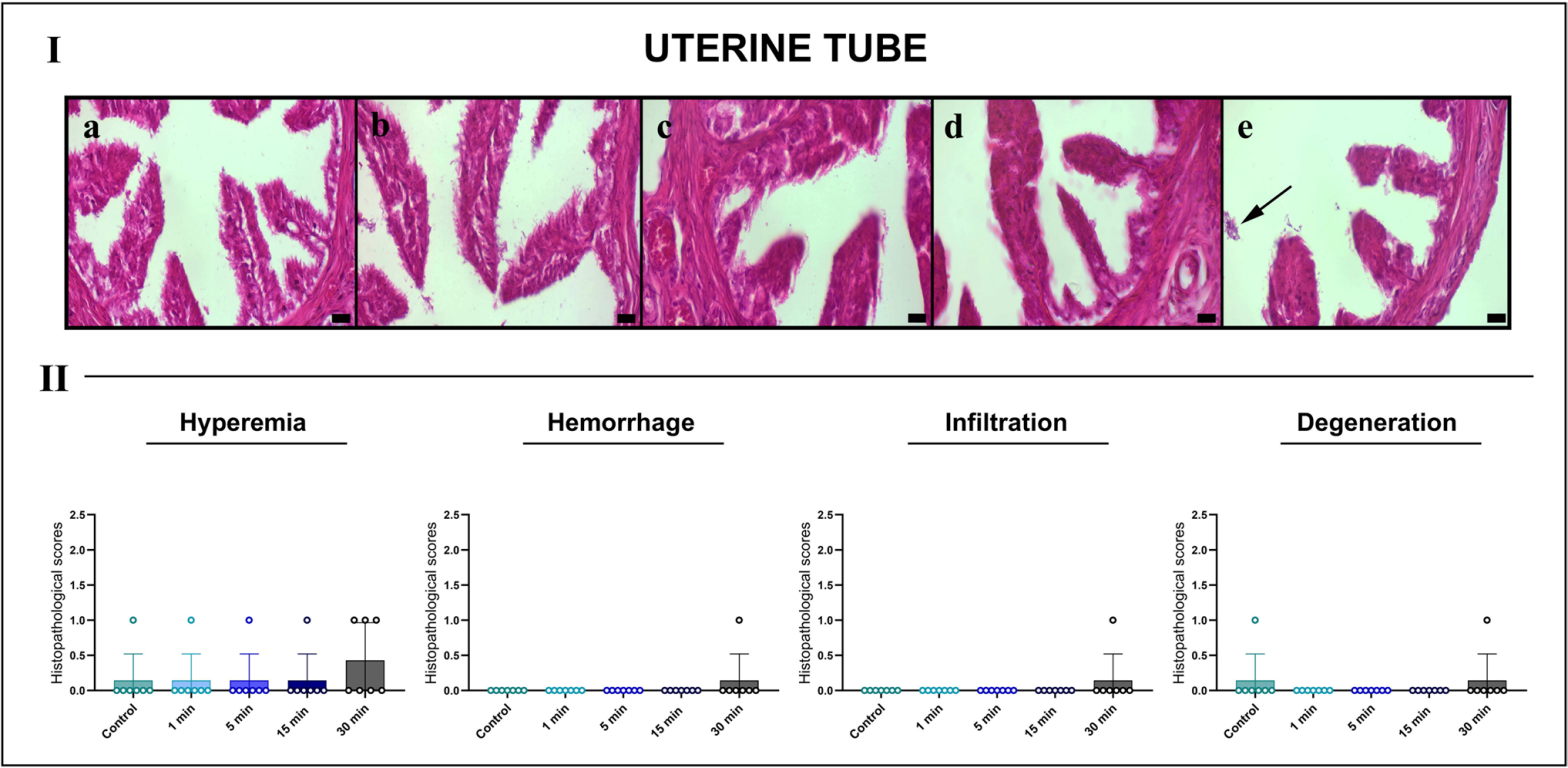
Representative HE-stained sections of the uterine tubes across experimental groups (I) and semi-quantitative histopathological scoring (II). **A** Control group displays intact mucosal folds, organized stroma and no detectable tissue damage. **B** 1-min and **C** 5-min exposure groups maintain normal epithelial and stromal architecture. **D** 15-min group shows mild epithelial flattening and slight hyperemia. **E** 30-min EF exposure results in focal hyperemia, early epithelial degeneration, and sparse cellular debris (arrow). Scale bars, 20 μm. Semi-quantitative scoring indicates a nonsignificant trend toward increased hyperemia and hemorrhage in the 30-min group, while infiltration and degeneration remain minimal. Scores were analyzed using Kruskal–Wallis test with Dunn’s multiple comparison post hoc test

Histological assessment of the uterine tubes showed indicated largely preserved structural integrity in the control, 1-min, and 5-min groups, with intact ciliated columnar epithelium and organized stromal architecture. The 15-min group exhibited only minimal epithelial flattening and mild stromal edema. In contrast, the 30-min exposure group, mild vascular congestion, focal subepithelial edema, occasional erythrocyte extravasation, and limited inflammatory cell infiltration were observed, along with early degenerative changes in epithelial folds. Despite these alterations, uterine tubes were comparatively more resistant to EF-induced damage than ovarian and uterine tissues. Semi-quantitative scoring revealed only mild, nonsignificant increases in damage parameters in the 30-min group (Fig. 4).

Uterine tissues exhibited a more pronounced response to EF exposure. The control group showed normal endometrial glandular and stromal architecture, without evidence of edema, hemorrhage, or inflammation. The 1- and 5-min groups maintained largely intact morphology with only minor, nonsignificant vascular changes. Beginning at 15 min, mild stromal edema and early epithelial distortion were observed. These alterations were markedly exacerbated in the 30-min group, which demonstrated prominent vascular congestion, focal hemorrhage, dense leukocyte infiltration, and degenerative changes in glandular epithelium, including cytoplasmic vacuolization and epithelial flattening.

**Fig. 5:**
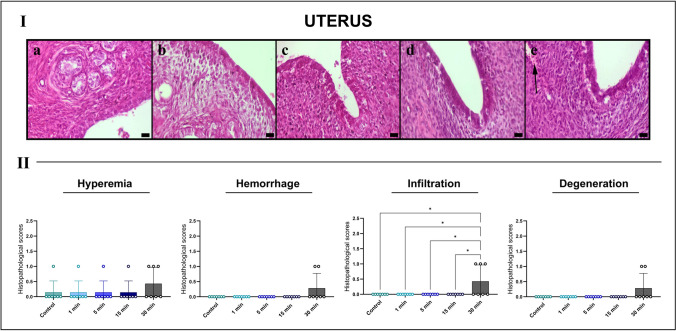
Representative HE-stained sections of rat uterine tissues across experimental groups (I), and semi-quantitative histopathological scoring (II). **A** Control group shows normal endometrial gland and stromal architecture. **B** 1-min and **C** 5-min exposure groups maintain intact epithelium and minimal stromal changes. **D** 15-min group exhibits mild hyperemia. **E** 30-min EF exposure induces focal hemorrhage (arrow). Scale bars, 20 μm. * Semi-quantitative scoring demonstrates a significant increase in inflammatory infiltration (*p < 0.05) in the 30-min group compared with all other groups, analyzed using Kruskal–Wallis test with Dunn’s multiple comparison post hoc test, while other parameters remain minimal

Histopathological scoring confirmed a significant increase in inflammatory cell infiltration (*p < 0.05) in the 30-min group compared with all other groups, highlighting inflammation as a dominant feature of uterine injury following prolonged EF exposure (Fig. 5).

### Immunohistochemical findings

**Fig. 6:**
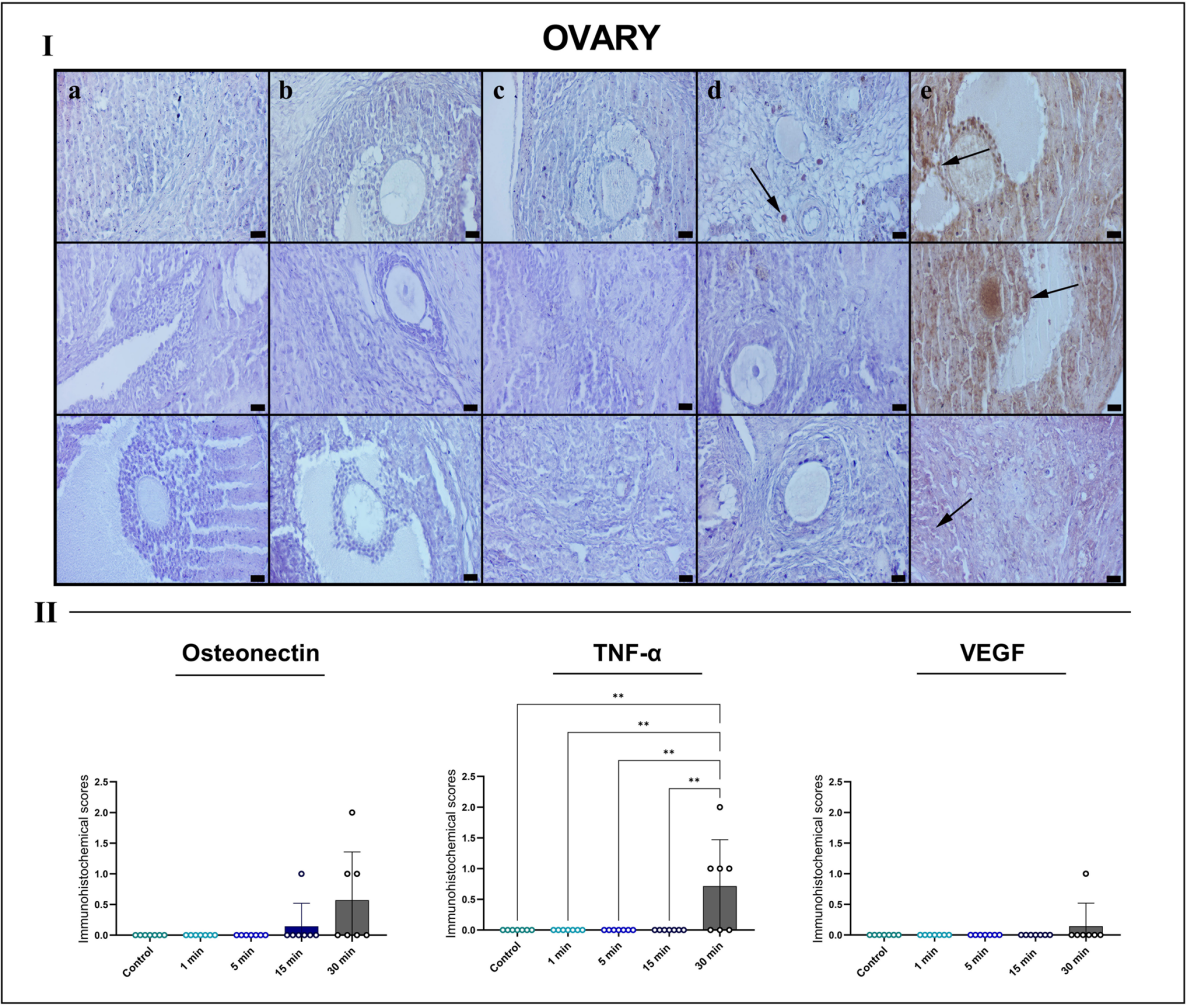
Immunohistochemical analysis of rat ovarian tissues for osteonectin (top row), TNF-α (middle row) and VEGF (bottom row) across experimental groups (I) with quantitative immunoreactivity scoring (II). Groups: **A** Control, **B** 1 min, **C** 5 min, **D** 15 min and **E** 30 min EF exposure groups. Minimal immunoreactivity is observed in the control and shorter exposure groups. In the 30-min group, strong osteonectin and TNF-α expression is detected in the stromal and follicular regions (arrows), while VEGF staining shows focal enhancement. Scale bars, 20 μm. Quantitative analysis indicates significantly increased TNF-α immunopositivity (**p < 0.01) in the 30-min group, with a nonsignificant upward trend toward higher osteonectin and VEGF expression. *TNF-α* tumor necrosis factor alpha, *VEGF* vascular endothelial growth factor

Immunohistochemical analysis of ovarian tissues demonstrated a clear upregulation of inflammation- and remodeling-related markers following prolonged EF exposure. In the control, 1-min, 5-min, and 15-min groups, osteonectin, TNF-α, and VEGF expression was minimal or absent. By contrast, the 30-min group exhibited robust osteonectin immunoreactivity, particularly within stromal regions, indicative of extracellular matrix remodeling. TNF-α expression was markedly increased in both follicular and interstitial compartments, reflecting an active inflammatory response. VEGF staining showed focal enhancement around dilated vessels and degenerative areas, consistent with angiogenic activation. Quantitative analysis confirmed significant increases in osteonectin and TNF-α expression, while VEGF showed a nonsignificant upward trend (Fig. 6).

**Fig. 7:**
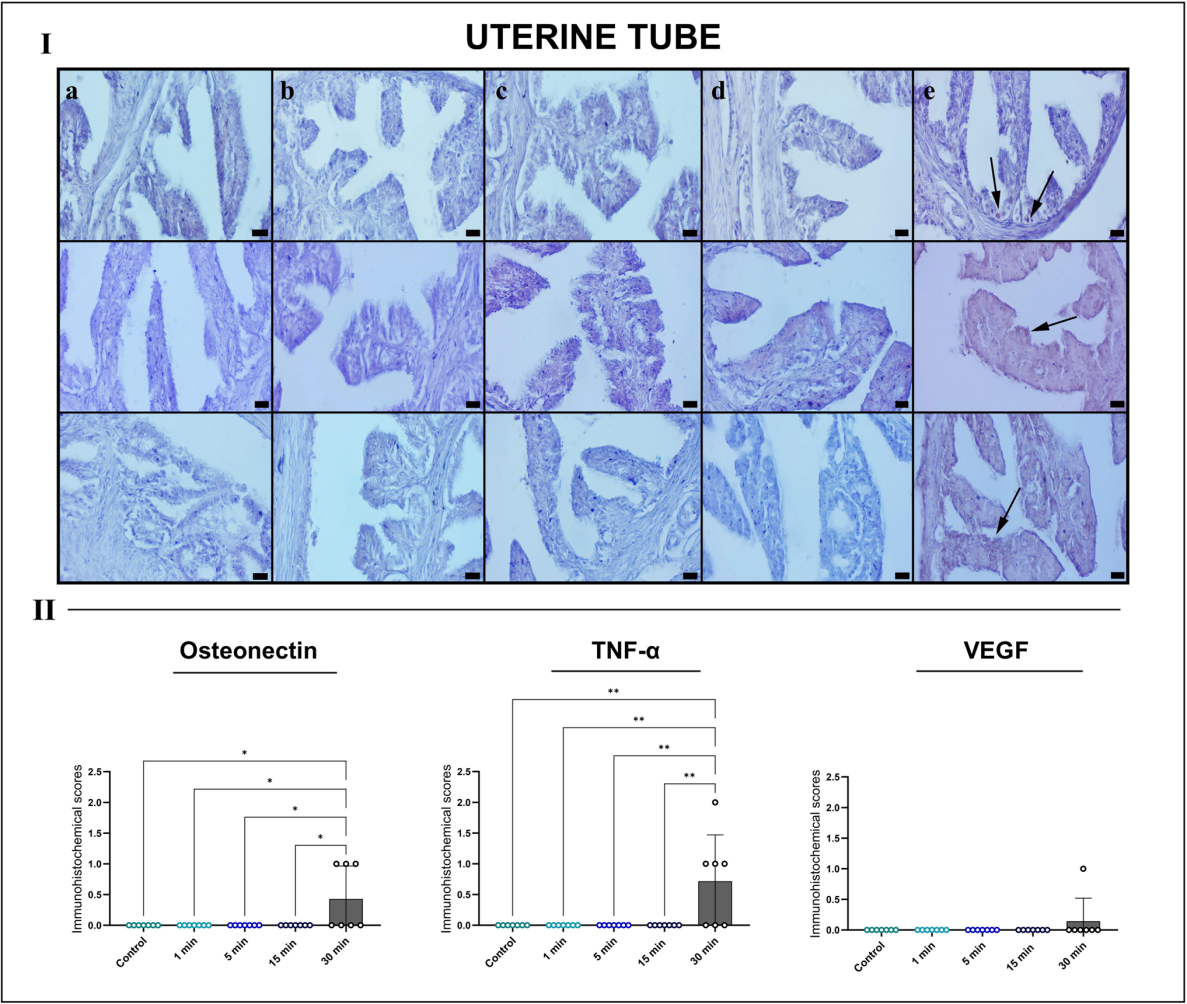
Immunohistochemical analysis of the uterine tube tissues for osteonectin (top row), TNF-α (middle row), and VEGF (bottom row) across experimental groups (I) with quantitative immunoreactivity scoring (II). Groups: **A** control, **B** 1 min, **C** 5 min, **D** 15 min, and **E** 30 min EF exposure. Minimal to absent staining is observed in groups **A**–**D**. The 30-min group (**E**) exhibits markedly increased osteonectin and TNF-α immunoreactivity in the stromal compartment (arrows), while VEGF shows focal endothelial positivity. Scale bars, 20 μm. *Quantitative scoring indicates significant increases in osteonectin (*p < 0.05) and TNF-α (**p < 0.01) in the 30-min group, whereas VEGF showed a nonsignificant upward trend. *TNF-α* tumor necrosis factor alpha, *VEGF* vascular endothelial growth factor

In uterine tubes, immunoreactivity for all three markers remained negligible in the control, 1-min, 5-min, and 15-min groups. However, the 30-min group displayed pronounced osteonectin and TNF-α upregulation, predominantly in the lamina propria and subepithelial stromal regions, indicating EF-induced tissue stress and inflammatory activation. VEGF expression showed focal vascular-associated positivity without reaching statistical significance. Quantitative analysis confirmed significant increases in osteonectin (*p < 0.05) and TNF-α (**p < 0.01) in the 30-min group (Fig. 7).

**Fig. 8:**
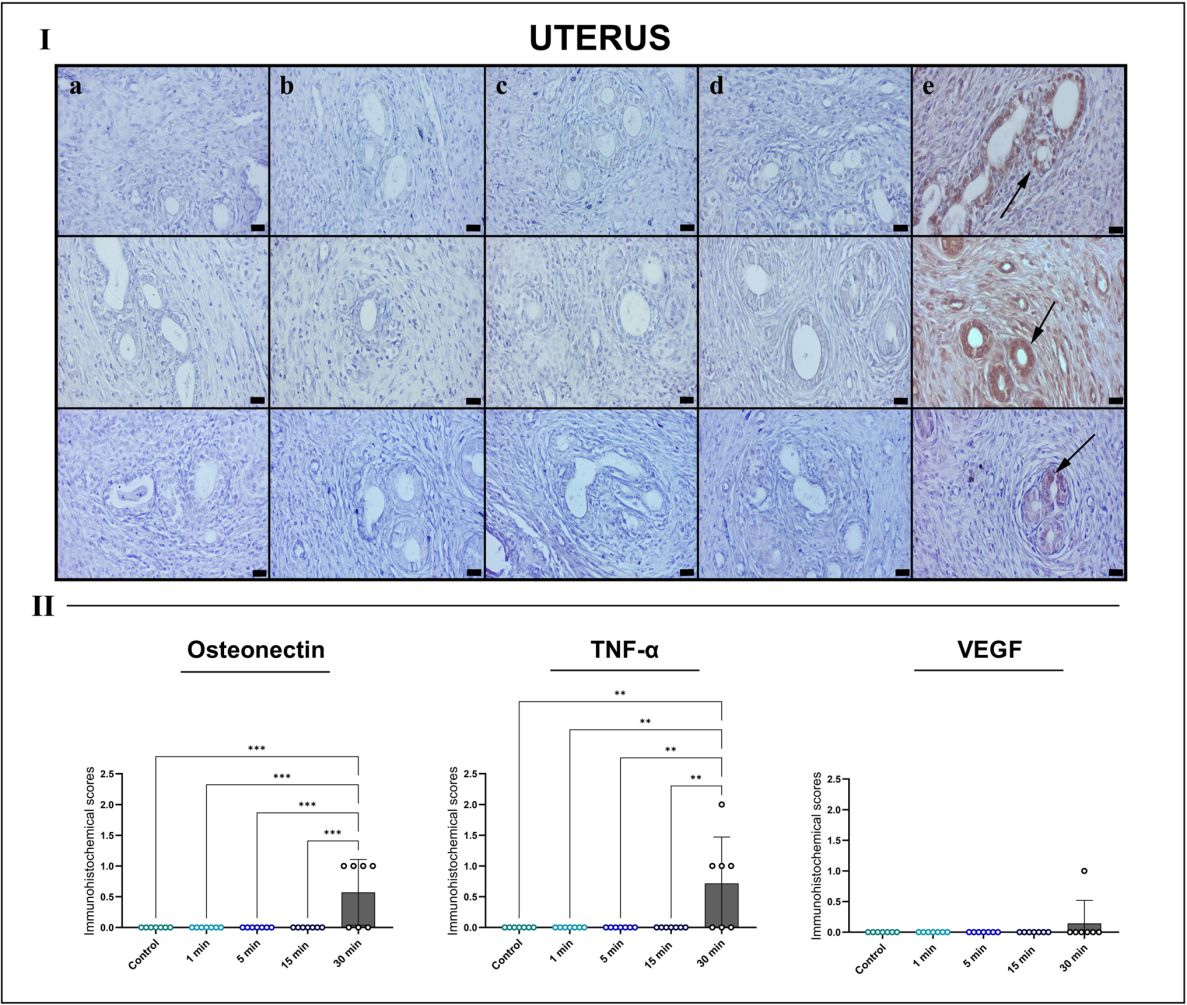
Immunohistochemical analysis of rat uterine tissues for osteonectin (top row), TNF-α (middle row), and VEGF (bottom row) across experimental groups (I) with quantitative immunoreactivity scoring (II). Groups: **A** control, **B** 1 min, **C** 5 min, **D** 15 min, and **E** 30 min EF exposure. Minimal immunopositivity is noted in groups **A**–**D**. In the 30-min group (**E**), TNF-α exhibits strong immunoreactivity in both epithelium and stromal compartments, while osteonectin and VEGF show focal positivity (arrows). Scale bars, 20 μm. *Quantification analysis reveals significantly increased osteonectin (***p < 0.001) and TNF-α (**p < 0.01) expression in the 30-min group, with nonsignificant upwards trends for VEGF. *TNF-α* tumor necrosis factor alpha, *VEGF* vascular endothelial growth factor

Uterine tissues exhibited the most prominent immunohistochemical response. While marker expression remained minimal in the control and shorter exposure groups, the 30-min group showed intense TNF-α immunoreactivity in both stromal and epithelial compartments. Osteonectin expression was significantly elevated in subepithelial and perivascular regions, indicating active matrix remodeling. VEGF positivity was observed focally around endometrial vessels but did not reach statistical significance. Quantitative analysis confirmed significant increases in osteonectin (***p < 0.001) and TNF-α (**p < 0.01) expression in the 30-min group (Fig. 8).

## Discussion

Although numerous studies have investigated the biological effects of EMFs (Oral et al. 2006; Jung et al. 2007; Roushangar and Rad 2007; Khaki et al. 2011; Roshangar et al. 2014; Alchalabi et al. 2016), research focusing specifically on high-intensity EFs remain limited. Most existing literature emphasizes magnetic or combined electromagnetic exposures, leaving the specific effects of high-voltage EFs on hormonally sensitive organs insufficiently characterized. The present study addresses this gap by providing a focused histopathological and immunohistochemical evaluation of EF-induced alterations in the female reproductive system.

The selection of a 10 kV/m EF intensity was guided by experimental and translational considerations. Fields of this magnitude have been shown to elicit measurable bioelectrical and cellular responses without causing overt thermal injury (Ozcan et al. 2025; Akın et al. 2025). Comparable localized field intensities may occur transiently in certain occupational and medical settings, including high-voltage installations and electrotherapeutic applications, in accordance with WHO and ICNIRP guidelines (WHO, 2007; ICNIRP, 2010; Yarmush et al. 2014). Although higher than typical environmental exposures, this intensity represents a realistic upper-bound scenario relevant to occupational health risk assessment.

Exposure to 10 kV/m induced organ-specific inflammatory, vascular, and extracellular matrix (ECM) remodeling responses in hormonally responsive reproductive tissues. Progressive histopathological alterations in the ovary and uterus—including hemorrhage, leukocytic infiltration, and epithelial degeneration—highlight the heightened vulnerability of metabolically active and endocrine-regulated organs. These structural changes were accompanied by marked upregulation of TNF-α and VEGF, indicating activation of coordinated inflammatory and angiogenic pathways consistent with established mechanisms of tissue injury and repair (Ferrara 2004; Malik et al. 2006; Aguilar-Cazares et al. 2019; Li et al. 2025).

The observed responses may also be contextualized within the broader framework of Tumor Treating Fields (TTFields). Studies by Kirson and colleagues demonstrated that externally applied electric fields can modulate cellular proliferation, membrane polarization, mitotic spindle organization, and intracellular signaling in a field strength– and frequency-dependent manner (Kirson et al. 2004, 2007). Although TTFields are typically delivered as alternating fields at optimized intermediate frequencies to target tumor cells, these findings underscore that the critical role of field intensity in determining biological outcomes.

Importantly, the present study did not aim to replicate the classical TTFields paradigm but to evaluate short-term, high-intensity EF exposure in non-neoplastic reproductive tissue in vivo. The consistent histological and immunohistochemical alterations observed demonstrate that 10 kV/m constitutes a biologically active yet experimentally controllable exposure capable of eliciting tissue-level inflammatory and angiogenic responses. By modulating vascular and inflammatory pathways beyond direct cytotoxic mechanisms, these findings advance the understanding of EF–tissue interactions and highlight the need for future dose–response studies to define threshold levels associated with reproductive tissue susceptibility.

Among the examined tissues, the ovary appeared particularly susceptible to EF exposure, exhibiting pronounced follicular degeneration and hemorrhage. This vulnerability likely reflects EF-induced perturbations of membrane potential, calcium homeostasis, and endothelial permeability, mechanisms previously linked to electrical stress responses (Blank and Goodman 2009; Okatan et al. 2018). Such primary disruptions may trigger secondary cascades involving oxidative stress and pro-inflammatory signaling, amplifying tissue injury. Uterine alterations—including stromal edema, epithelial flattening, and inflammatory infiltration—further support activation of cytokine-mediated innate immune pathways. The robust TNF-α immunopositivity observed suggests engagement of NF-κB–dependent signaling, which regulates both inflammatory and apoptotic responses under cellular stress (Malik et al. 2006; Blank and Goodman 2009).

Although uterine tubes displayed relatively mild histological alterations, the significant upregulation of osteonectin and TNF-α indicates that molecular stress responses may precede overt morphological injury. Osteonectin, a matricellular protein involved in ECM remodeling and stress adaptation, appears to function as an early indicator of EF-induced subclinical tissue stress. Its consistent elevation across reproductive tissues aligns with previous reports of osteonectin activation under mechanical, oxidative, or electromagnetic stress (Bradshaw and Sage 2001; Calleja-Agius et al. 2009; Herrera et al. 2009).

VEGF immunoreactivity localized predominantly to perivascular and damaged regions, suggesting a compensatory angiogenic response to microvascular injury. The modest and occasionally nonsignificant increases in VEGF expression may reflect its delayed activation relative to inflammatory signaling, as angiogenesis typically follows the initial inflammatory phase of tissue repair (Malik et al. 2006; Arablou et al. 2021). Longer exposure durations or extended post-exposure intervals may be required to capture peak VEGF dynamics.

Previous studies have shown that EMF exposure can enhance ROS generation through mitochondrial dysfunction and redox imbalance (Feng et al. 2016; Consales et al. 2021). Although ROS levels were not assessed in the present study, a similar mechanism may contribute to EF-induced tissue injury. Collectively, these findings support a mechanistic model in which high-intensity EF exposure disrupts membrane electrochemical gradients, induces endothelial injury, and triggers TNF-α–mediated inflammation, followed by osteonectin- and VEGF-driven ECM remodeling and angiogenic responses. While the absence of direct oxidative stress and DNA damage measurements represents a limitation, the strong correlation between histopathological injury and immunohistochemical marker expression provides a robust foundation for future mechanistic and longitudinal studies addressing reproductive function and recovery following EF exposure.

Another important methodological consideration is that animals were not physically restrained during EF exposure. This approach was deliberately adopted to minimize stress-induced neuroendocrine and inflammatory responses that could independently affect reproductive tissue integrity and confound EF-related effects. Although physical restraint can reduce movement-related variability, it is well established that restraint stress activates the hypothalamic–pituitary–adrenal (HPA) axis and elevates circulating glucocorticoids, which may directly influence vascular reactivity and inflammatory pathways (Herman et al. 2016). Therefore, a nonrestrictive exposure paradigm was preferred to reduce stress-related bias and to better approximate real-life environmental and occupational exposure conditions. Future studies directly comparing restrained and nonrestrained models would be valuable to delineate the relative contributions of movement and stress to EF-induced biological responses.

Furthermore, the uterus is a highly dynamic organ that undergoes cyclic morphological and molecular changes throughout the estrous cycle. Endometrial thickness, vascularization, inflammatory cell distribution, and angiogenic mediator expression fluctuate according to hormonal stage (Jain et al. 2022). Although physiological cycle-dependent variation may have contributed partially to the observed histological and immunohistochemical findings, animals were randomly allocated to experimental groups and maintained under identical environmental conditions, which likely distributed estrous-related variability evenly across groups. Importantly, the consistent and exposure-dependent pattern of inflammatory and angiogenic alterations in EF-exposed animals supports the interpretation that the detected changes are predominantly attributable to high-intensity EF exposure rather than normal cyclical variation.

Finally, although comprehensive multi-point spatial mapping of the electric field was not performed across the entire cage volume, field intensity was verified at representative points within the central exposure region using a surrogate load to approximate the dielectric properties of the animals. Measured values showed minimal variation around the nominal 10 kV/m intensity, supporting the assumption that animals located in the central portion of the apparatus experienced comparable field conditions. Because animals were unrestrained and free to move during exposure, these measurements should be interpreted as nominal field values characterizing the central exposure zone rather than a fully resolved spatial field distribution. Future studies incorporating detailed three-dimensional field mapping would further refine exposure characterization and enhance dosimetric accuracy.

### Study limitation

Several limitations of the present study should be acknowledged. First, functional reproductive assessments—such as hormonal profiling, systematic monitoring of the estrous cycle, and fertility evaluation—were not performed. Although high-intensity EF exposure elicited clear structural and molecular alterations in reproductive tissues, integration of endocrine and functional outcome measures would provide a more comprehensive understanding of the in vivo reproductive consequences.

Second, estrous cycle synchronization was not performed before EF exposure. Uterine morphology, vascularization, inflammatory activity, and angiogenic signaling fluctuate physiologically across the estrous cycle, and the absence of cycle staging may have introduced biological variability. While random allocation of animals to experimental groups was used to reduce systematic bias, future studies incorporating vaginal cytology-based estrous determination or synchronization protocols would allow tighter hormonal control and more precise interpretation of uterine-specific EF effects. Such methodological refinement would further enhance the translational relevance of the findings.

Third, time-matched sham exposure groups were not included for each exposure duration. Although control animals were maintained under identical environmental conditions, the inclusion of duration-matched sham controls in future experiments would help to definitively exclude potential stress- or handling-related confounding effects.

Finally, detailed spatial mapping of electric field within the exposure region was not performed. Although exposure parameters were standardized and nominal field intensities were verified at representative points using a surrogate load, quantitative mapping under loaded conditions would improve reproducibility and provide more precise characterization of tissue-level field exposure. Addressing these methodological considerations in future investigations will strengthen causal inference and clarify the biological impact of high-voltage EF exposure on reproductive tissues.

## Conclusions

The present study indicates that short-term exposure to a nominal high-intensity electric field (10 kV/m), verified at predefined measurement points within the exposure system, is associated with tissue-specific alterations in female reproductive organs. These alterations were characterized by increased inflammatory signaling, angiogenic activity, and extracellular matrix remodeling. Among the biomarkers, osteonectin demonstrated a particularly responsive expression pattern, showing increased immunoreactivity even in regions where overt histopathological injury was not prominent. This observation suggests that osteonectin may serve as a sensitive molecular indicator of early tissue stress responses associated with electric-field exposure, although further mechanistic studies are required to confirm its role as an early biomarker.

From a translational perspective, these findings highlight the importance of further investigating the potential biological effects of localized high-intensity EFs, particularly in hormonally responsive reproductive tissues. While the present experimental model does not directly replicate occupational exposure conditions, the observed molecular and histopathological responses suggest that reproductive tissues may represent a biologically responsive target in EF exposure scenarios.

Overall, the study provides preliminary mechanistic insight into tissue responses associated with short-term EF exposure and underscores the need for future investigations incorporating comprehensive exposure characterization, quantitative field mapping, and additional molecular endpoints to more fully define the biological and safety implications of high-voltage electric-field environments.

## Data Availability

The data used in the study are included in the manuscript.
